# An Evaluation of the Aboistop Citronella-Spray Collar as a Treatment for Barking of Domestic Dogs

**DOI:** 10.5402/2011/759379

**Published:** 2012-01-23

**Authors:** Rebecca J. Sargisson, Rynae Butler, Douglas Elliffe

**Affiliations:** ^1^School of Psychology, University of Waikato, Private Bag 3105, Hamilton 3240, New Zealand; ^2^Behavioural Consultant, 52 Otumoetai Road, Brookfield, Tauranga 3110, New Zealand; ^3^Department of Psychology, The University of Auckland, Private Bag 92019, Auckland 1142, New Zealand

## Abstract

The aim of our study was to investigate whether citronella-spray collars offer a humane alternative to electric-shock collars to reduce the barking of domestic dogs. The Aboistop collar was applied to seven dogs with problematic barking behaviour by the dogs' owners in a series of case studies concurrently run. Vocalisation of the dogs was recorded in the problem context under baseline conditions, inactive collar conditions, and active collar conditions. The Aboistop collar was effective at reducing problem vocalization for only three of seven dogs and appeared to be most effective for dogs whose problem barking had developed more recently. The collar may be more humane than other punishment methods, but it did produce stress reactions which varied in severity across the dogs. *Clinical Relevance*. In our study, the collar was applied by the dogs' owners in order to test whether the collar would be effective when used by members of the public. While the results here are preliminary, they suggest that the collar may be effective for some dogs, but not for others, when applied by dog owners for the treatment of problem vocalisation. Further research is required to determine whether the collar could be effective when administered by a trained professional.

## 1. Introduction

Punishment is a commonly used modifier of human and animal behaviour because it has been shown to be effective at reducing unwanted behaviour. For example, aversive conditioning can be an effective method of eliminating the vocalising of dogs [1]. The effectiveness of punishment increases with its intensity (e.g., [2]), and punishment is more effective if it is introduced abruptly at moderate intensity rather than faded in from low intensity [3]. However, the ethical implications of imposing punishment can lead to a desire to use less severe forms of punishment. Dog owners, in particular, are warned that physical punishment should never be used [4], and that, if used, punishment should be delivered remotely to avoid association of the owner with the aversive stimulus [4, 5].

Antibark collars, as a form of remote punishment to reduce the vocalization of dogs, have several advantages. They are capable of delivering different forms of aversive stimuli, for example, electric shocks or high-frequency sounds. Because the shock is triggered by vibrations from the dog's larynx when the dog barks, punishment can be delivered consistently, and immediately, after the response, rather than haphazardly, or after a delay. Temporally contingent consequences are more effective at modifying behaviour than delayed punishment [6]. Additionally, punishment delivered by an antibark collar is unconnected with the owner, reducing the likelihood that the owner will become a conditioned aversive stimulus that the dog learns to fear or become aggressive towards [7]. However, high intensity shocks may be overwhelming or cruel, causing skin lesions [8], chronic stress [9, 10], fear and pain responses [11, 12], and even death [13].

For humane reasons, dog owners are likely to prefer less intensely punishing solutions to their dogs' barking behaviour. In one study, dog owners reported a preference for citronella-, or lemon-spray collars, which deliver a spray of citronella when a microphone detects barking, over shock collars, because they perceived them as more humane [14]. No significant difference was found in mean plasma-cortisol values (a stress indicator) between dogs that wore shock versus lemon-spray collars [15], and it is not clear whether lemon-spray collars result in fewer pain and stress reactions than shock collars.

However, previous research has shown that low-intensity punishment, such as that delivered by a citronella collar, may be less effective than intense punishment [2]. Seven of nine dog owners in Juarbe-Diaz and Houpt's [14] study reported reductions in frequency, intensity, and duration of their dogs' barking when they wore citronella collars for a 2-week period. These dog owners were more satisfied overall with citronella collars than with shock collars. Steiss et al. [15] found lemon-spray collars to be just as effective as shock collars in eliminating barking. The dogs' barking was completely eliminated by the third day of wearing either a shock collar, or a lemon-spray collar, with both collar types being equally effective. Steiss et al. [15], however, did not evaluate whether the improvement was maintained over longer periods although they stated that there was no evidence of habituation when the dogs wore the collars intermittently over a 2-week period.

Moffat et al. [16] also found citronella collars to be effective in reducing the barking of 77% of the dogs who received a 5 min application of the collar in a veterinary hospital. The severity of barking was rated subjectively, however, and no followup was carried out to determine whether improvement was maintained.

**Figure 1:**
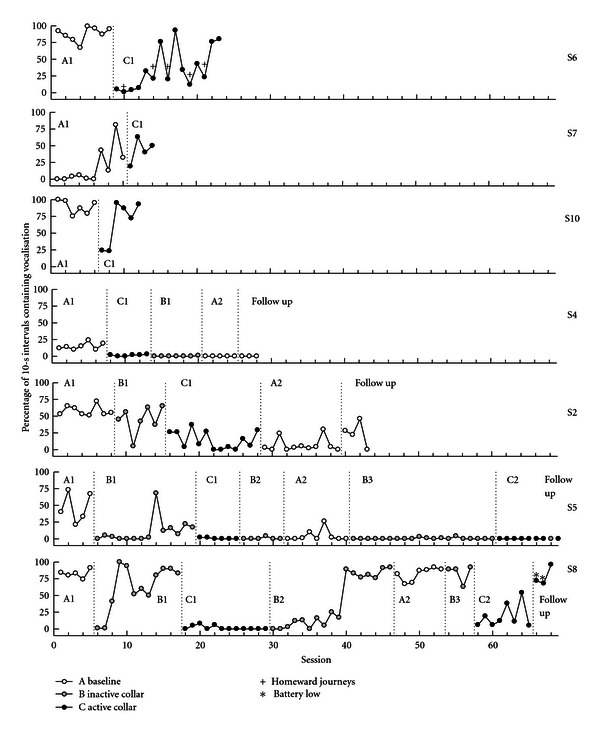
Percentage of 10-s intervals containing vocalizations across sessions for each dog. Vertical lines reflect condition breaks for each dog.

Wells [17] showed a greater reduction in the barking of dogs that wore citronella spray collars intermittently (30 min every other day) than for dogs that wore the collars continuously (30 min every day) over a 3-week period. After a large initial decrease in the reported frequency of barking, barking frequency increased for both groups of dogs, but more quickly for dogs exposed more regularly to the collar. After the 3-week treatment period, and 1 week without collar use, the frequency of barking had returned to pretreatment levels for dogs exposed to the collar every day in treatment. Barking frequency remained lower than pretreatment levels for the intermittent group but had not stabilised, and appeared to be still subject to an upward trend [17, Figure  1]. Wells [17] did not attempt to measure behavioural reactions of the dogs to the collars (such as stress or fear), and the efficacy of the treatment was based on subjective owner ratings of the frequency of barking.

We had two main aims in addition to measuring the efficacy of mild punishment delivered via citronella collars in reducing vocalization of domestic dogs. Firstly, we set out to measure the longer-term effectiveness of citronella collars, by measuring vocalization across multiple treatment sessions, and 3 months after the end of the treatment phase. We sought to obtain a more objective measurement of barking than in previous research, by tape recording the dogs' vocalizations. Secondly, we monitored the dogs for any signs of distress while using the collars.

## 2. Method

### 2.1. Subjects

Ten dogs were selected from veterinary referrals or phone calls to the second author. Dogs weighing less than 5 kg, or with previous experience of the citronella collar, were excluded. A description of the dogs' characteristics is shown in Table 1. All dog owners had received an official warning, a complaint from neighbours, or reported that their dog's vocalizing reduced the quality of the dog's relationship with the owner. The data from three dogs (S1, S3, and S9) are not shown here because their baseline levels of vocalization were extremely low prior to the application of the collar.

Ethics approval was received from the University of Auckland's Animal Ethics Committee. Free treatment was offered to dog owners in exchange for participation.

### 2.2. Apparatus

The Aboistop collar contains a laryngophone which activates a spray jet via a selector. According to the manufacturer, the spray is activated by a 95 dB-level bark. However, A-weighted testing in the psychophysics laboratory of the University of Auckland indicated that the spray was triggered by an 80 dB sound. The collar is powered by a 6 V battery which lasts for an estimated 1000 sprays. A reservoir contained within the casing of the collar, and which sits on the dog's throat, holds approximately 20 sprays of citronella. Upon activation, a single spray of citronella is forced upward towards the dog's mouth and nose upon each separate bark. Thus, a continually barking dog will experience multiple sprays. The canister makes a pressure release sound similar to a can of fly spray when activated.

 Data were collected by videotape (with a VHS GF-450 JVC video camera) for Dog S7 and audiotape (with a Bush C5100s) for all other dogs.

### 2.3. Conditions and Design

The study was designed as a series of case studies run concurrently. Thus, the conditions, and their order, were selected according to the needs of each dog and its owner.  Figure 1 shows the order of conditions and the number of sessions completed by each dog in each condition.

In “A”, or baseline, conditions, the Aboistop collar was not applied. In “B” conditions, vocalizations were recorded while some dogs wore an inactive Aboistop collar. The inactive collar did not produce any spray or noise concomitant with vocalisation. This condition was included where the vocalization problem was nonurgent in order to test the effect of the introduction of a novel stimulus in the absence of citronella. Condition B1 was omitted with dogs whose owners sought a faster resolution of their dogs' problem vocalization. “C” conditions involved placing an active Aboistop collar on the dog.

Owners received instruction on how to operate and test the collar and initiated the recording device for the duration of the session. The second author was present for the first three C1 conditions for all dogs to ensure that owners were applying the collar correctly. All applications of the collar were recorded. That is, the dogs did not wear the collar in unrecorded periods. Every instance in which the dog was exposed to the problem context was recorded. During the treatment phase, the dogs wore the collar in every problem context. The second author contacted owners frequently to ensure they were applying the collar correctly, and recording every instance in which the dog was exposed to the problem context.

The treatment was applied as it would be by a novice pet owner who had purchased the collar. That is, if the citronella canister emptied completely during a session, it was not refilled until the next session because, in standard use, the owner would be unlikely to be present to refill it. Similarly, any problems encountered during treatment, such as the battery running low, or non-compliance with instructions, were assumed to be representative of typical owner use of the device.

Condition C1 was terminated for Dogs S6, S7, and S10 after 15, 4, and 6 sessions once it was determined that the collar did not effectively reduce their vocalization. Active collars were used for a maximum of two weeks for the remaining dogs. When vocalization appeared to be eliminated under active collar condition (C1), the collar was inactivated (B2) in an attempt to generalise behaviour control so that the collar could eventually be removed without recurrence of the vocalization.

A session began with a cue identified by the dog owner as a trigger for the dog's vocalization. The mean duration (and standard deviation) in minutes of sessions was 11 (6), 60 (0), 60 (0), 19 (15), 72 (26), 9 (11), 12 (8) for Dogs S2, S4, S5, S6, S7, S8, and S10. Three dogs (S4, S5, and S7) vocalised when home alone, so session length was determined by the usual length of the owners' absence. Session length was set at 60 min for Dogs S4 and S5 and was usually 60, 90, or 120 min for S7. Session length necessarily varied for the other four dogs because they vocalised during car journeys. Thus, the duration of each session matched the duration of the car journey.

Follow-up sessions were conducted three months after the final session for Dogs S2, S4, S5, and S8. Follow-up sessions were identical to those in prior conditions. Dogs S2 and S4 conducted the followup under baseline conditions because their vocalizations were successfully reduced and the collar was no longer required. Dogs S6, S7, and S10 did not participate in follow-up sessions because the collar was not effective at reducing vocalization, and treatment was discontinued.

### 2.4. Behavioural-Reaction Test

The second author recorded behavioural reactions during the first three sessions of Condition C1. Thereafter, reactions were monitored by the dog owner, and regular home visits were made by the second author to assess the dogs' well being. The criteria for behavioural reactions were largely based on those observed in the studies of Piette [18] and Brunelat [11]. Operational definitions are presented in Table 2.

### 2.5. Data Analysis

The second author used a partial-interval recording method to quantify the prerecorded data. A stopwatch was used to time each interval and record the duration of each session. For each 10-s interval, any occurrence of a vocal response was noted with a tick and the absence of a vocal response with a cross. A vocal response was defined as howling, whining, or barking. For each session, frequency of vocalization was the percentage of 10-s intervals that contained vocalizations. The percentage of each session that contained vocalisations was analysed both graphically and statistically (see below).

 Reliability checks were conducted on a random 10% of sessions across all phases. A psychologist trained to use the recording method recorded the presence or absence of vocalizations, separately, but at the same time, as the primary observer. Overall agreement between observers was 95%.

## 3. Results

Figure 1 shows the percentage of 10-s intervals containing vocalization for each dog for each session. Conditions are displayed in the order completed (reading left to right).

Using a graphical analysis, for two (S5 and S8) of the three dogs whose baseline condition (A1) was followed by an inactive collar condition (B1), vocalization was reduced by the application of the inactive collar. For S8, vocalization was quickly restored to baseline levels in B1.

The citronella collar appeared to be effective at eliminating vocalization for Dogs S4 and S5 and reducing vocalization for S2. The effect generalised to collar-absent situations for all three dogs although vocalization was never completely eliminated for S2.

The collar was ineffective for the remaining four dogs, with vocalizations increasing to baseline levels over time. Dog S6's vocalizations were lower for homeward journeys (marked with a + in Figure 1) than for outgoing journeys, showing that the collar was not effective in all situations.

Using a statistical analysis, a paired *t*-test showed that mean vocalisations were significantly lower during the first session with the active collar applied (C1; mean = 7.85) compared to the mean for the last baseline session (A1; mean = 39.69; *t*(9) = 2.26, *P* = .01). However, a paired *t*-test comparing the mean for the last baseline session with the mean of the last C1 session for each dog (mean = 35.50) was no longer significant (*t*(9) = 2.26, *P* = .52). These results suggest that the initial reduction in vocalisation produced by the active collar was not maintained with continued use.

 Dogs for whom the citronella collar appeared effective at eliminating or reducing vocalization (S2, S4, and S5) were characterised by a shorter vocalization history (mean = 5.33 months, SD = 5.77, min⁡ = 2, max⁡ = 12 months) than those dogs for whom the collars were not effective (S6, S7, S8, and S10; mean = 24 months, SD = 12.96, min⁡ = 12, and max⁡ = 42 months).

During exposure to the citronella collar, dogs commonly froze, shook their heads, sneezed, and jumped backwards. These responses were fleeting and disappeared with extended exposure. Dog S5 showed serious distress reactions, hiding under a veranda and trembling continuously during the latter sessions of Condition C1. These responses immediately disappeared when the collar was removed and never recurred.

## 4. Discussion

The results indicate that, when the Aboistop collar was applied by dog owners, it was effective for three of the seven dogs, with vocalisation completely eliminated for two dogs, and reduced for one.

For two of the three dogs (S5 and S8) that experienced an inactive collar condition (B1), vocalisation was reduced upon the application of the inactive collar. For Dog S8, this reduction was transient. The initial reduction for S8 may have been due to this dog's previous exposure to a shock collar. Simply introducing a novel stimulus, however, can reduce responding [7]. Indeed, given that four dogs did not experience the inactive collar condition (B1), the initial reduction in vocalization seen for Dogs S6, S7, and S10 when the active collar was applied (C1) may also be due to the introduction of a novel stimulus, suggesting that the active collar itself may never have been effective for them.

The Aboistop collar appeared more effective for dogs with a shorter history of vocalisation. Although we cannot conclude that the collars are ineffective on dogs with a long history of excessive vocalization, the implication is interesting. In practice, owners whose dogs have a short history of vocalization problems are probably less likely to use antibark collars, as dog owners will tend to seek nonaversive methods before aversive ones to remedy the behaviour problems of their dogs.

Several owners found the collar oversensitive to extraneous stimuli. Head-shaking, vigorous movement, and panting sometimes resulted in inappropriate activation of the collar. Brunelat [11] also observed problems with the sensitivity of the collar. This noncontingent application of punishment may be another factor contributing to its ineffectiveness. The collar not only released citronella in the absence of barking but also failed to release citronella following some vocalizations. Further research on anti-bark collars, which could be conducted under artificial conditions, should investigate the reliability of the relationship between vocalization and activation of the collar.

One owner experienced a malfunction when the collar suddenly exploded and left a dent in the ceiling of his living room. The manufacturer, Dynavet France, reported that 70 of 100,000 collars sold by them had also exploded (Dynavet, pers. comm., November 10, 1995). Luckily, the collar was sitting on a table and not being worn by the dog or held by the dog owner, at the time.

Distress reactions associated with the activation of the collar suggest that a spray of citronella was only mildly aversive to most dogs and were linked to the spray's novelty. However, the citronella collar was clearly more aversive for some dogs than for others.

Indifference to the citronella collar appeared to develop in Condition C1 for Dogs S6 and S10 and Condition C2 for Dog S8. The owners of these dogs observed that the collar was drained of citronella due to continuous vocalization. Thus, in conditions of normal use, the citronella collar may quickly lose its effectiveness if vocalization occurs at a high rate.

Given that stronger punishment is more effective [7], it may be that citronella collars do not sufficiently punish problem vocalizations. Dog owners may perceive citronella collars as more humane than shock collars [14] but should be aware that a low-intensity punisher, such as the citronella anti-bark collar, is less likely to be effective at reducing the problem behaviour than stronger punishment [3]. Using any level of punishment via an anti-bark collar to reduce the vocalizations of dogs is ethically concerning, particularly when the problem behaviour occurs in the owner's absence, as there is no one present to ensure that the collar is not causing undue suffering.

In summary, while the small sample size precludes unequivocal evidence of the efficacy of citronella collars, it appeared effective for three and ineffective for four of the seven dogs. Thus, citronella collars may be effective for reducing or eliminating the vocalisations of some dogs but not others. In our study, it appeared that the collars were more effective with dogs that had a shorter vocalisation history. For other dogs, an initial reduction in vocalisation when the active collar was applied was not maintained with continued use.

## Figures and Tables

**Table 1:** Summary of characteristics of each dog (breed, age at beginning of study, sex, desexing status, age dog was acquired by its owner, hypothesised cause of the problem vocalisation, duration of the problem, type of vocalisation, and the context in which the problem vocalisation occurred).

Dog	Breed	Age (mth)	Sex	Neutered	Age acquired (wk)	Cause	Duration (mth)	Type	Problem context
S1	Bichon frise	18	F	Y	8	Territorial	9	Bark	Home
S2	Bull terrier cross	24	M	N	8	Excitability	12	Whine	Car
S3	Dalmation	8	M	N	8	Boredom	4	Bark	Home alone
S4	German shepherd	6	M	N	7	Excitability	2	Bark	Home
S5	Bull terrier cross	24	F	N	6	Boredom	2	Bark	Home alone
S6	Huntaway cross	24	F	N	6	Excitability	18	Bark	Car
S7	weimaraner	132	F	N	6	Boredom	12	Bark	Home alone
S8	Border Collie	30	F	Y	6	Excitability	24	Whine	Car
S9	Borzoi	60	M	Y	0	Territorial	49	Bark	Home
S10	Cocker spaniel	48	M	Y	36	Excitability	42	Bark	Car

**Table 2:** Operational definitions of behavioural reactions based on those observed in the studies of Piette [18] and Brunelat [11].

Behaviour	Definition
Hiding	Subject uses a physical structure to conceal itself (e.g., under a bed or table).
Aggression	The display of typical defence reflexes such as biting, growling, or attacks towards animate or inanimate object.
Momentary inhibition	Subject remains still for a few seconds without showing any other behavioural reaction.
Escape	Subject attempts to run away from the location in which the spray was released.
Sneezing	Subject sneezes once or several times immediately after the discharge of citronella.
Trembling	Noticeable trembling.
Prostration	Lying down.
Head shaking	Subject briskly shakes its head from left to right as it generally does after a bath.
Other	Other unspecified behaviours are reported as they appear.
